# Scientia Potentia Est: How the Italian World of Oncology Changes in the COVID-19 Pandemic

**DOI:** 10.1200/GO.20.00209

**Published:** 2020-07-07

**Authors:** Zelmira Ballatore, Lucia Bastianelli, Filippo Merloni, Nicoletta Ranallo, Luca Cantini, Giulia Marcantognini, Rossana Berardi

**Affiliations:** ^1^ Azienda Ospedaliero Universitaria Ospedali Riuniti di Ancona Umberto I G M Lancisi G Salesi, Ancona, Italy; ^2^ Universita Politecnica delle Marche, Ancona, Italy

## Abstract

**PURPOSE:**

After coronavirus disease 2019 (COVID-19) was declared a pandemic by the WHO, a response from the Italian Health System to react to an unprecedented condition became necessary and sudden. The COVID-19 pandemic has required oncologists to redefine clinical organization and patient management. The purpose of our study was to document the difficulties emerging during the SARS-CoV-2 pandemic in Italian oncology.

**METHODS:**

We broadcasted an electronic survey to oncologic health care professionals. It consisted of 45 questions ranging from individual perception of pandemic management by hospital centers to physicians’ and nurses’ psychological distress and patient care.

**RESULTS:**

A total of 383 oncology health workers participated in the survey. The majority were female (71.8%) and from central Italy (46.2%). Impressively, a total of 357 (93%) participants declared the oncologic department reorganized routine clinical activity, but only 40.5% were adequately trained about the required procedures; 20% of the survey respondents think they have not received adequate and timely protective devices.

**CONCLUSION:**

Our survey demonstrated the flexibility of oncologic teams. However, the emergency response quality has been heterogeneous, and several drawbacks have emerged from the first analyses investigating how the world of oncology changes in the COVID-19 pandemic. Information, protection, testing, and training of health care professionals are key words that should be kept in mind to encourage recovery after this tragedy and to be ready to face a similar emergency in the future.

## INTRODUCTION

The spread of coronavirus disease 2019 (COVID-19) has been declared pandemic by the WHO, having infected > 100,000 people in 100 countries.^1^ At present, 212 countries are affected, with 1,439,516 confirmed cases of infection and 85,711 deaths.^2^

**CONTEXT**

**Key Objective**
How did Italian oncology departments deal with difficulties that emerged during the COVID-19 pandemic?
**Knowledge Generated**
This electronic survey was answered by 383 oncologic health care professionals. A total of 357 participants declared the oncologic department reorganized routine clinical activity, but only 40.5% were adequately trained about the required procedures; 20% of the survey attendees think they have not received adequate and timely protective devices.
**Relevance**
The Italian National Health System must improve its reaction time in terms of health worker training and protection in anticipation of similar future emergency.

Since March 8, 2020, several extraordinary decree laws have been activated in Italy, with important restrictive measures to minimize movement of people and social activities, aimed to reduce the probability of transmission and contagion. Although at present the infection rate seems to have peaked, the decrease in spreading is turning down at a slow pace, with already > 140,000 confirmed cases and deaths close to 20,000 .^3^

The COVID-19 pandemic is pressing the Italian National Health System because of shortage of hospital beds, intensive care unit beds, ventilators, and personal protective equipment (PPE); the pandemic is also affecting the availability of the medical workforce, because doctors and nurses are becoming ill or quarantined.^3^ This aspect already proved a dramatic factor in Wuhan, where 41% of the COVID-19 cases resulted from hospital-related transmission.^4^

With such evidence, protecting health care workers is paramount,^5^ but taking into account the rapidly growing imbalance between supply and demand for medical resources, the question of how they can be wisely allocated during the emergency arises.

To date, in Italy, we do not know what the rate of infected health workers is, but we know that among those SARS-CoV-2–positive professionals the average age is younger than that of the general population (49 instead of 63 years), and the proportion between men and women is reversed, with 35.8% being male.^6^

In this highly complex health system scenario, the clash between treatment for patients with cancer and COVID-19 management requirements is clear. The question is not whether to set priorities but how to reorganize treatment of patients with cancer in a consistent and ethical approach.^7^

Patients with cancer are generally very susceptible to infections, and these are known to be a major cause of death in this setting. The greatest vulnerability is mainly linked to 3 factors: underlying disease, anticancer treatments, and comorbidities.^8,9^

The purpose of our study was to document the current state of Italian oncology and to evaluate the difficulties that emerged in the management of patients with cancer during the SARS-CoV-2 pandemic.

## METHODS

Between March 18 and April 9, 2020, we attempted to survey oncologic health care professionals (physicians and nurses) working in the Italian National Health Care System.

The survey consisted of 45 multiple choice questions, including 4 demographic questions (Table 1); 7 questions regarding clinical activity reorganization; 9 questions about the individual perception of resources, information, and staff training management by hospital centers (Table 2); 11 questions concerning patient management (Table 3); and 14 questions concerning health care professionals’ psychological distress (Table 4). No open-ended questions were provided.

**TABLE 1:**
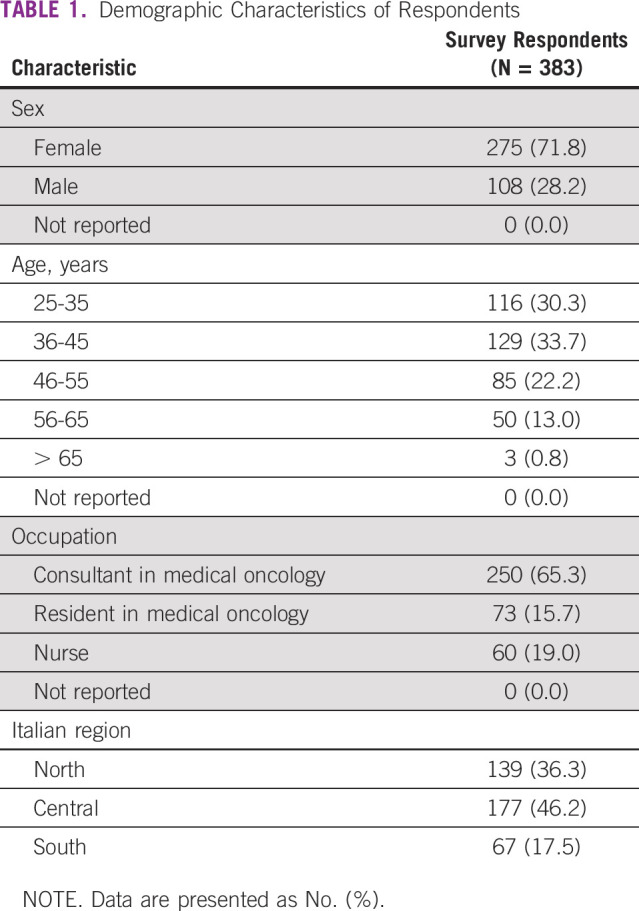
Demographic Characteristics of Respondents

**TABLE 2:**
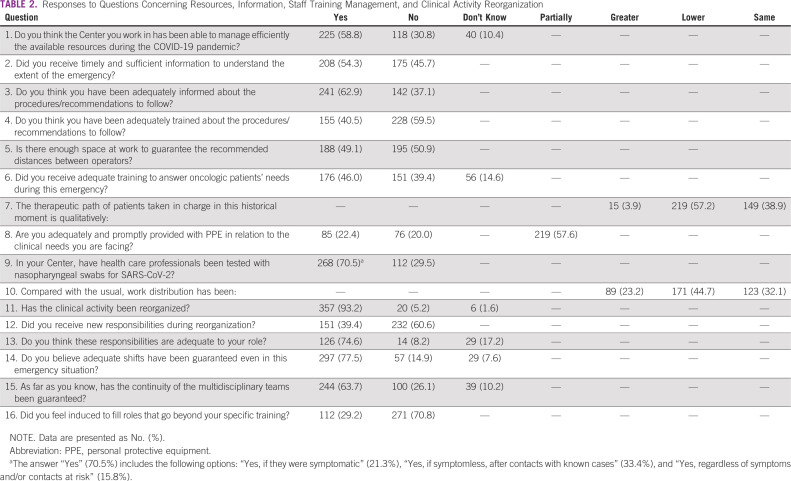
Responses to Questions Concerning Resources, Information, Staff Training Management, and Clinical Activity Reorganization

**TABLE 3:**
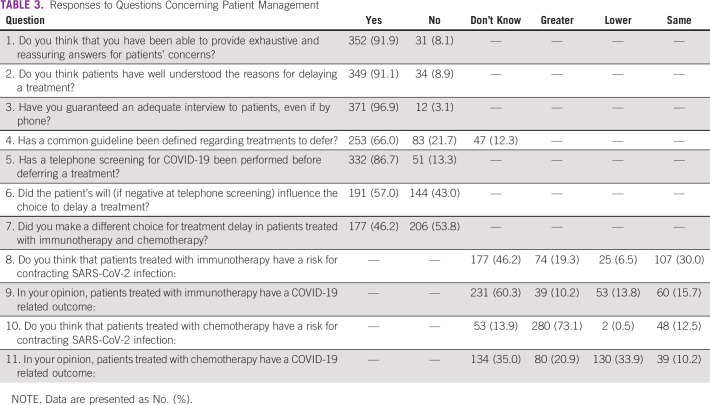
Responses to Questions Concerning Patient Management

**TABLE 4:**
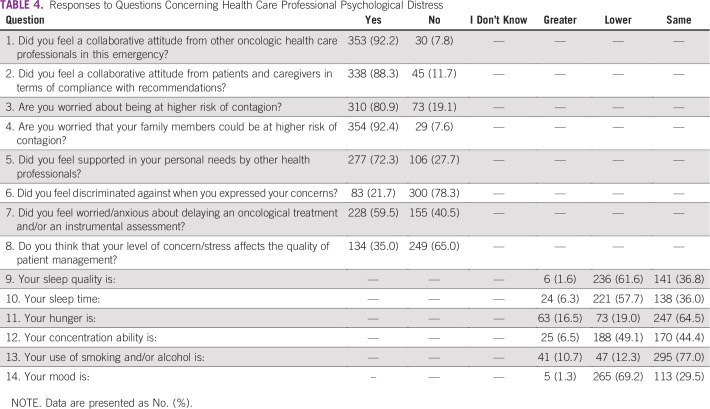
Responses to Questions Concerning Health Care Professional Psychological Distress

The survey was entirely electronic and anonymous (Google Forms) and was broadcasted to oncology health workers by mailing contacts, word of mouth, and social networks.

The survey was promoted by Clinica Oncologica, AOU Riuniti di Ancona-Università Politecnica delle Marche. According to Italian law (resolution March 1, 2012, Gazzetta Ufficiale n.72 of March 26, 2012), ethics approval was not required for the current study.

## RESULTS

A total of 383 oncology health workers participated in the survey. The majority of respondents were between 36 and 45 years old, female (71.8%), from central Italy (46.2%), and medical oncologists (65.3%; Table 1).

During the COVID-19 pandemic, the major requirement concerned oncological activity reorganization; 93% of respondents declared that the oncologic department reorganized routine clinical activity, although 60.6% said they did not receive new responsibilities (Table 2).

Among all participants, 225 (58.8%) subjects stated that their site organization managed the available resources during the COVID-19 pandemic efficiently; 54.5% received timely and sufficient information to understand the extent of the problem.

Most of the respondents (62.9%) were adequately informed about the procedures/recommendations, and 40.5% were adequately trained about the required procedures/recommendations. In maintaining safe distances between health care workers, approximately 1 out of 2 (50.9%) did not have adequate space available to comply with this recommendation (Table 2).

On the emergency-led shortage of PPE, 20% of the survey attendees think they have not received adequate and timely protective devices with respect to clinical needs, and according to 57.6%, the supply of these devices was only partial (Table 2).

The survey revealed serious critical issues regarding screening: 29.5% of professionals were not subjected to swabs, 21.3% performed screening only if symptomatic, 33.4% if asymptomatic after contact with known infected cases, and only 15.8% at least once, regardless of symptoms or contacts (Table 2).

In this dramatic historical moment, patients taken in charge have an inevitably modified therapeutic pathway, with a provided quality of care defined as lower (57.2%) than the standard by health care workers. However, multidisciplinary tumor board meetings were guaranteed in 63.7% of cases by alternative communication channels (for example Skype, video calls, etc; Table 2).

The COVID-19 pandemic determined the need to reschedule oncologic treatments, and 34% of health care professionals answered that they do not have or know a defined common guideline. In 86.7% of cases, a phone screening to patients allowed the choice to defer a treatment (Table 3).

Among the 250 oncologists who filled out the survey 41.6% and 56.4% did not know if the contagion risk and the infection outcome could be modified by the receipt of immunotherapy, respectively. On the other hand, the majority of oncologists define chemotherapy as a potential risk factor for the infection (69.2%), and 36.8% believe that chemotherapy may negatively affect outcome.

More than 80% (310 out of 383) of interviewees said they feel worried about being at greater risk of contagion than the general population; 92.4% feared transmitting the virus to family members. Deferring treatments has caused fear/anxiety in 228 of those interviewed (59.5%).

Symptoms of the stressful situation emerged, with a deterioration in sleep quality in 61.6% of professionals (57.7% sleep less than usual), a worsening of mood (69.2%), and lower concentration ability (49.1%), with consequent perception of lower quality of patient management by the majority (59.5%) of oncologic workers (Table 4).

## DISCUSSION

This is the first survey, to our knowledge, that investigated individual perception of resources and training, clinical reorganization, patient management, and burnout in oncologic health care professionals during the COVID-19 emergency in Italy. Considering that the Italian Oncology Association (AIOM) counts approximately 2,500 members, our sample of 383 participants may be representative of the country.

The first major emerging data are that approximately 60% of respondents assessed their department organization as managing the available resources effectively, and 63% of respondents received timely and sufficient information about the emergency and procedures/recommendations; the downside is that 40% were not.

These are important data if we consider that less than half (40.5%) were adequately trained, and almost all professionals work in an oncology department that was forced to reorganize clinical activity. It is mandatory to remember that hospital staff should receive necessary technical training to perform their tasks better.^10^

In addition to the not-negligible lack of adequate training, the survey reveals impressive gaps in terms of health protection, a scenario already described.^11^ It is probable that the lack of training and of PPE, in addition to the emergency per se, led 80% of professionals to a very high perception of risk and the consequent concern of infectious danger for their family members. Times like this can leave physicians stranded between commitment to the community and responsibility to their families.^12^

Furthermore, the survey revealed a lack in the execution of the testing swab; 30% of professionals did not perform it, causing the failure to recognize asymptomatic carriers allowing diffusion of the virus. Nevertheless, health care worker screening is crucial to contain the infection.^13^

Our survey demonstrated the high flexibility required for oncologic teams to reorganize their daily routines.^14^ Identifying patient management needs and multidisciplinary reorganization were specifically set up, but unfortunately they seem to be inadequate substitutes for traditional management. Most of the oncologists declared their perception of a qualitatively lower treatment than the period before the pandemic, and they feel fear and anxiety deferring treatments.

National and international oncologist associations (AIOM, the European Society for Medical Oncology, and ASCO) proposed guidelines for patients with cancer in the COVID-19 emergency, but many unanswered questions are raised even more dramatically at this time: Which is the correct patient selection? Which treatments to continue and which to stop? What timing? Which choices and which ethics?^15^

These questions are even more important for oncologists who work in a public health system like the Italian one. One of the most current questions is the effect of immunotherapy on infection risk and outcome, and almost half of survey respondents were unaware of this issue. There are some ongoing initiatives and studies aimed to answer this question and understand the real impact of COVID-19 in patients with cancer, such as TERAVOLT.^16^

Among the main psychological consequences in health care professionals, sleep quality was worse in more than half of interviewees, 70% declared a deterioration in mood, and half of them reported lower concentration than usual. These results showed that oncologic workers are at great risk to develop post-traumatic stress symptoms. Our findings are similar to those obtained by literature data published during the SARS outbreak in 2004.^17^ Also, a Chinese mental health survey of medical staff during the COVID-19 pandemic, highlighted that anxiety and stress disorders were frequent.^18^ These data suggest health professionals’ mental health assessment, support, and treatment are crucial tools to guarantee the required standard of cancer therapy during the COVID-19 pandemic.^19^

In Italy, on January 31,^20^ the prepandemic state was declared, but very little was done to be ready, to correctly inform citizens, to train health workers, and to prepare protection plans for hospitals and health workers; it is possible that what was happening in China was enormously underestimated. Perhaps without previous direct experiences, it was foreseeable that the Italian National Health System was generally not sufficiently prepared for pandemic events.

The emergency response quality has been heterogeneous,^21^ and this has determined several critical issues in specialized, already critical patient–oriented environments such as oncology departments, considering the importance of the need to adequately prepare for the allocation of scarce resources before it becomes necessary. Italian oncologists demonstrated resilient skill to face emergencies ensuring the continuum of cancer care that is the cornerstone of oncology.^22^

There are several key issues that are obviously borderline health and politics–related subjects.

**FIG 1:**
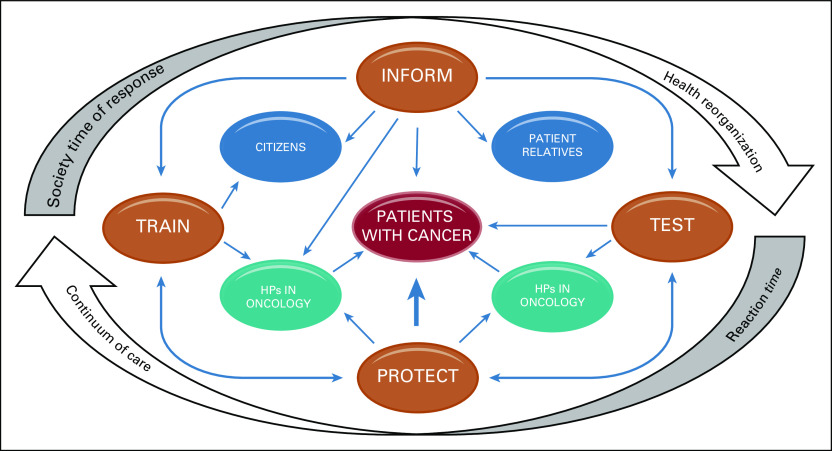
Fundamental elements to ensure the continuum of cancer care during a pandemic. HPs, health care professionals.

Citizen information and society expected timely response, health organization preparation, and reaction time and further protection measures for the weakest, most demanding subject classes, such as oncology patients (Fig 1).

The most critical considerations still relate to the entry of the virus into hospitals, the heart of health care, where citizens are treated and where patients with cancer are protected. Instead, the oncologic department had to limit access to care to protect patients from a more dangerous disease for them, witnessing a paradox of health care.

The protection and isolation of key health workers, the support of training, family life, and, most important, psychological aspects, have all proven blind spots, which the survey has captured in the case of the Italian National Health systems. These will have to be food for thought for several lines of management in both politics and health system direction to encourage recovery after this tragedy and to be ready to face a similar emergency in the future.
